# Folic Acid Supplement Intake and Risk of Colorectal Cancer in Women; A Case Control Study

**DOI:** 10.5334/aogh.2664

**Published:** 2020-02-27

**Authors:** Sara Moazzen, Saeed Dastgiri, Roya Dolatkhah, Hossein Mashhadi Abdolahi, Behrooz Z. Alizadeh, Geertruida H. de Bock

**Affiliations:** 1 Department of Epidemiology, University Medical Center Groningen, University of Groningen, Groningen, NL; 2 Health Services Management Research Center, Tabriz University of Medical Sciences, Tabriz, IR; 3 Liver and Gastrointestinal Diseases Research Center, Tabriz University of Medical Sciences, Tabriz, IR

## Abstract

**Background:**

An ongoing controversy exists on the role of folic acid supplementation in colorectal cancer risk among epidemiological studies.

**Objective:**

To assess the association between maternal folic acid supplementation and colorectal cancer risk.

**Methods:**

A paired matched case control study of 405 subjects was performed, including women residing in 135 villages of East Azerbaijan, Iran. Per area, subjects were followed regularly in local healthcare centers, where health- and social-related information have been collected prospectively in face to face interviews by well-trained health workers. We extracted folic acid supplement intake, baseline characteristics, and confounders from healthcare records. The data for study participants were linked to national cancer registry repositories, from which we retrieved the data of 135 women diagnosed with colorectal cancer between 2005 to 2015. Two hundred seventy controls were individually matched with cases in terms of residing village, age, and gender. We applied multivariate conditional logistic regression to estimate odds ratios (ORs) and 95% confidence intervals (CIs).

**Findings:**

There was no significant association between folic acid supplementation and colorectal cancer risk in those with history of folic acid intake compared to those with no history of intake (OR 0.95; 95% CI 0.59 to 1.53), in those with less than five years of folic acid (0.79; 0.45 to 1.39) or in those with ≥5 years intake (1.09; 0.52 to 2.26). This risk did not change after adjustment for covariates or further stratification.

**Conclusions:**

Maternal folic acid supplementation did not affect colorectal cancer risk in a population where supplemental folic acid is prescribed with regular intervals for women of child-bearing age.

## Introduction

Folic acid supplementation is widely prescribed to women of child-bearing age due to its determinative role in preventing most prevalent congenital anomalies and neuronal tube defects [1]. To increase the efficacy of folic acid supplementation, fortification of staple foods with folic acid has also been legislated in numerous states across the world [2]. As a consequence, overloads of folate in body have been demonstrated by epidemiological studies [3,4], raising concerns given the possible role of folic acid in colorectal cancer promotion by stimulating precancerous cell growth if administrated in early stages of cancer [5].

Findings on folic acid/folate intake and colorectal cancer among nations with different ethnicities and lifestyles are inconclusive [6]. While a large investigation of 10 European countries, as well as those from US and Japanese populations, did not support an association between plasma folate and risk of colorectal cancer [7,8,9], one study on women residing across the US demonstrated a highly protective effect of high folate levels in blood [10]. Other studies conducted among Chinese and Swedish people reported an adverse effect of high folate levels in blood on the risk of colorectal cancer [11,12,13]. In our previous investigation, we found no effect of folic acid supplementation on the risk of colorectal cancer in a meta-analysis of 35 studies [6]. The contraversies in findings among different nations are partly due to applied methods in assessing folic acid effects, such as measuring red blood cell folate, or plasma folate, or total folate intake [6], or variation in ethnicity and life style [14]. Further, given the findings are mainly from developed nations and the data from developing regions who have different lifestyles, and that environmental and genetic background are neglected, it is crucial to have corresponding evidence from less represented regions with different ethnicities.

Given the controversies and lack of evidence from developing nations with less represented ethnicities, we aimed to investigate the effects of folic acid supplementation on the risk of colorectal cancer in an ethnically homogenous Turkish-Azari population, who were followed for a long time during and after the history of use of folic acid supplementation.

## Methods

In a paired matched case-control design, the association between folic acid supplementation intake and colorectal cancer risk in women was assessed.

### Setting

The study was performed in rural parts of East Azerbaijan, Iran. The area was chosen due to its having a mono ethnic background, mostly identical lifestyle, and the fact that the study province is among the regions with high rates of colorectal cancer (with an age standard rate of 13.32 per 100,000 in women, compared to a lower 5.34 per 100,000 incidence rate among women residing in other provinces of the country). These factors, combined with rigorous implementation of folic acid supplementation, make the population of this province suitable for investigating the effect of folic acid supplementation on colorectal cancer [15,16]. The detailed health data of women residing in rural areas are constantly recorded by well-trained health workers in local health, including the data related to antenatal, perinatal, and postnatal care, and supplementation and medical history.

### Population

**Figure 1:**
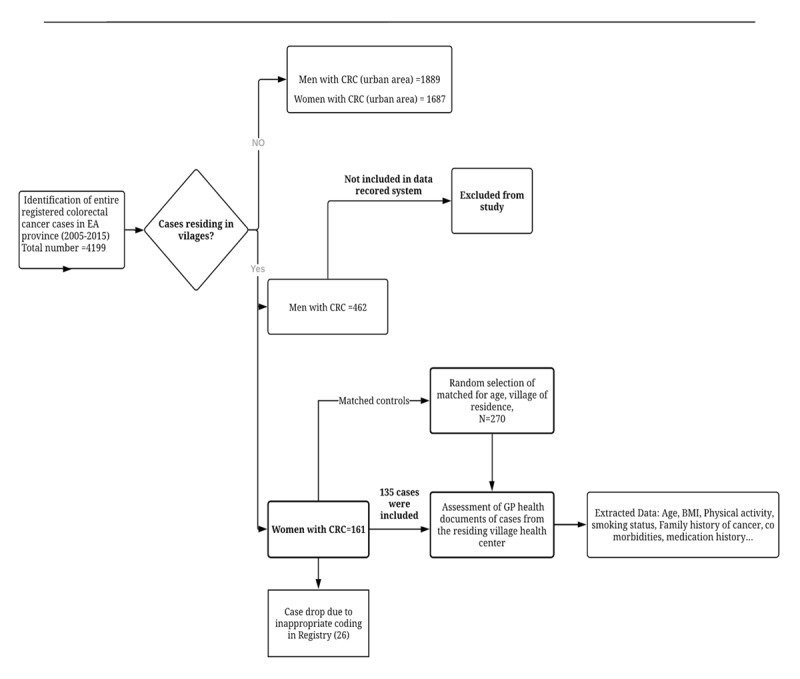
Study Flow chart. **Abbreviations:** CRC: Colorectal cancer, EA: East Azerbaijan, GP: General practitioner.

The study population was selected from women residing in entirely rural parts of East Azerbaijan province, Iran. A total of 178,867 women of 40–80 years age live in rural parts of this province. The cases and controls recruitment process is depicted in Figure 1. Pathologically confirmed colorectal cancer cases, according to ICD-OC 153.0–153.9 for colon and 154.0–154.8 for rectal cancer, diagnosed between 2004 and 2015, were identified from the National Cancer Registry repositories, using the linking identification numbers. Completeness of coverage was measured as the number of reported cases of cancer per year divided by the number of gastrointestinal tract cancers in Iran estimated by the WHO [17]. This demonstrated a 60–70% coverage of all cancer cases [18]. We identified 161 women who were diagnosed as having colorectal cancer. Twenty-six cases were excluded due to incorrect international disease classification coding in the cancer registry. Given the possibility of missed cases due to systematic errors or insufficient coverage of the cancer registry, the pathological records of all cancerous patient colorectal cancer among study participants. Using systematic random sampling methods for each case from the same village, two controls were selected who were alive and had not been diagnosed with any type of cancer. Controls were matched to cases based on residing village, age (within a range of 4 years), and sex. The protocol of the study was according to the Helsinki Declaration and was approved by ethical committee of Tabriz University of Medical sciences, Iran (Reference number: TBZMED.REC.1394.1193).

### Data collection

The data collection was based on registered data of folic acid supplement intake in health centers in villages. The dose of folic acid for supplementation was 2.8 mg/wk or 0.4 mg/day. The supplementation dosage and duration is explained in WHO guidelines [19]. Folic acid supplements are regularly prescribed for women of child-bearing age since past 12 years in villages. The supplement intake was not mandatory, and unconsumed supplements or refusal to take the supplements was recorded. This routine practice is implemented across the entire country using a structured data collection by digital method. Inhabitants of these regions are regularly visited, and detailed health related information on medications (including supplement intakes) and health status have been continuously recorded by well-trained health workers since 1991. Basic demographic data and certain lifestyle data assessments are recorded, including every prescription issued by the general practitioner, all consultations with the general practitioner, test results, diagnoses from primary and secondary care, and referrals to outpatient clinics, hospital admissions, and deaths. The data from cases and control health documents includes height, weight 5 years before cancer diagnosis, supplement intake, education, and marital status. Also included were lifestyle factors, such as smoking, physical activity, co-morbidities, medicine intake history, and duration of supplement intake, and were extracted using a structured questionnaire. In the data extraction process, one investigator (SM) was responsible for extracting the data from the digital healthcare system. The accuracy of extracted data was checked by a second investigator (RD). In the case of discrepancies, the main document was checked in the presence of a third author (MA).

### Data Analysis

Cases and controls were described regarding the characteristics using a q square test. These characteristics were included in the study based on a review of the literature [10,20]. The duration of data recording was calculated by the time period between the subjects’ initial recording date in the healthcare system and the date of cancer diagnosis for cases and their mached controls. We used conditional logistic regression using village and town level strata to estimate odds ratios (ORs) and 95% confidence intervals (CIs) for cancer in three subgroups of folic acid supplement intake (no history of intake, less than five years intake and more than five years intake) and for two tumor sites subgroups (colon and rectum). Using an evaluation test, the variables which modified the estimated risk for colorectal cancer by >%10 were identified. In the next step, in a multi-variate model, the estimated ORs for colorectal cancer in subgroups of folic acid supplement intake were adjusted for variables, including: smoking, body mass index, hypertension, diabetes, and GI disorders. In the next step, in order to assess the effect of time between folic acid intake and colorectal cancer incidence, the analysis was restricted to folic acid intake at least 36 months before the date of colorectal cancer diagnosis. Next, complete case analysis was performed by including cases and controls with complete data on adjusting variables in multivariate analyses. We conducted analyses stratified by factors that previously have been reported to affect or modify the association between folate intake and colorectal cancer risk, including age group defined into two groups (40–49 years and ≥50 years); history of vitamin D supplement intake defined as no intake versus history of intake; GI discomfort defined as no history of GI discomfort and history of GI discomfort; and hypertension history with no history of hypertension versus a history of hypertension. Data analysis was done using the clogit command of the survival package in R version 3.5.1.

## Results

**Figure 2:**
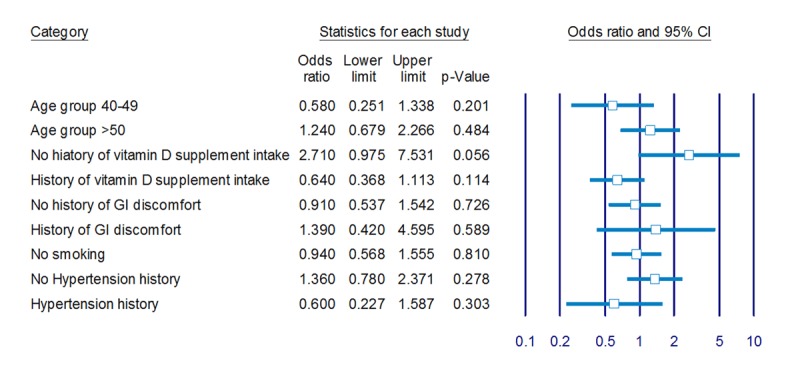
Odds ratio of colorectal cancer in cases with history of folic acid intake, compared with those with no history of intake, categorized by various factors.^1^ ^1^ Results are from multivariate conditional logistic regression for study population stratified by corresponding variables. Results for current smoking subgroups were not present due to small numbers.

The study population included 135 women with colorectal cancer and a total of 270 matched controls. See Table 1 and **Supplementary Table S1** for characteristics of the study population. Overall, cases had a mean age at diagnosis of 62.00 (SD 13.74) years. The cases were more likely to have a history of other GI disorders than were their matched controls (21.50% in cases compared to 9.60% in controls), whereas smoking status was similar for cases and controls. 23.70% of the study population (23.00% of cases and 24.10% of controls) had a history of folic acid intake in the past (Table 1). The mean observation period was identical for cases and their matched controls, and the mean duration of prospective data recording was 30.7 years (SD 12.57). The registered mean duration using folic acid supplement intake was 1.91 years (1.82 SD) in total, and for those users with more than five years, it was 5.8 years (1.59 SD). For the large majority of cases with folic acid supplement intake, the first intake within the follow-up period was at least 36 months before the data collection (87.10% cases; 93.80% controls). There was no significant association between folic acid supplementation and colorectal cancer risk (OR 0.95, 95% CI 0.59–1.53) for those with history of folic acid intake compared to no history of intake. The decreased risk of colorectal cancer in folic acid intake for less than 5 years was not significant (0.79; 0.45 to 1.39). The increased risk for colorectal cancer in using folic acid for longer than five years was not significant (1.09; 0.52 to 2.26). Folic acid intake was associated with 1.28 (0.70 to 2.14) times increase in risk of colon cancer and 1.42 (0.65 to 3.03) times decrease in rectal cancer. None of the observed results were significant (Table 2). Multivariate analysis and restricting analysis to history of intake to over 36 months since diagnosis showed minimal effects on estimated risk for colorectal cancer (Table 3). Associated risk also did not vary significantly between groups of patients stratified by age, history of vitamin D intake, gastro-intestinal disorders, smoking status, and hypertension (Figure 2).

**Table 1:** Baseline characteristic of study population, all variables stated as N(%)^1^.

Characteristics^2^	Cases (n = 135)	Controls (270)	P-value

Age (years) at index date			0.21
<40	9(6.7)	24(8.9)	
40–49	46(34.7)	71(26.2)	
50–59	73(54.1)	145(53.7)	
≥60	7(5.2)	30(11.1)	
BMI			0.68
<25.0	39(28.9)	94(34.8)	
25–29.9	47(34.8)	86(31.9)	
≥30	43(31.9)	65(24.1)	
Physical Activity			0.13
Normal	80(63.5)	188(70.4)	
Medium	35(27.8)	66(24.7)	
Intensive	11(8.7)	13(4.9)	
Smoking status			0.38
Never	120(88.9)	246(91.1)	
Past	6(4.4)	13(4.90)	
Current	1(0.7)	8(3.0)	
Alcohol^3^			0.48
Never	94(98.9)	260(99.6)	
Current	1(1.1)	1(0.4)	
Folic acid supplement intake			0.52
No intake	101(74.8)	204(75.5)	
In past	31(23.0)	65(24.1)	
<5 years intake	21(15.6)	51(18.8)	
≥5 years intake	10 (7.4)	14(5.2)	
Family history of cancer			0.11
No	98(74.8)	220(81.8)	
Yes	33(25.2)	49(18.2)	
Hypertension	33(24.4)	121(44.8)	0.00
Diabetes	10(7.4)	19(7.0)	0.89
GI discomfort	29(21.5)	26(9.6)	0.00
History of change in weight^4^			0.00
Yes	48(35.5)	22(10.37)	
No	72(64.4)	242(89.6)	
Vitamin D supplement intake			0.00
No history of intake	68(51.5)	46(17.0)	
History of intake in past	64(48.5)	224(82.9)	
<5 years intake	29(22.0)	40(14.8)	
≥5 years intake	35(26.5)	184(68.1)	


^1^ Q square test was applied to obtain the results. ^2^ Missing values (n, %), BMI (3, 0.1), Physical activity (12, 3.0), Smoking (11, 2.7), Alcohol (11, 2.7), Folic acid supplement intake (4, 1.0), Family history of cancer (5, 1.2), Hypertension (12, 3.0), Vitamin D intake (3, 0.7). ^3^ Past alcohol intake was not applicable to any of the participants. ^4^ Defined as >10% change (both increase and decrease) in body weight within study period. **Abbreviations:** BMI, body mass index; GI, gastrointestinal.

**Table 2:** Association between folic acid supplememnt intake and colorectal cancer risk^1^.

Folic acid Supplementation	Cases/control (n)	OR (95% CI)^2^

Intake status in past		
No history of intake	101/204	ref.
History of folic acid intake	31/65	0.95 (0.59 to 1.53)
No history of intake	101/204	ref.
<5 yrs intake	21/51	0.79 (0.45 to 1.39)
≥5 yrs intake	10/14	1.09 (0.52 to 2.26)
Classification by tumour site		
No history of intake	101/204	ref.
Colon (with history of folate intake)	23/65	1.28 (0.70 to 2.14)
Rectum (with history of folate intake)	8/65	0.71 (0.33 to1.53)


^1^ Adjusted for matching variables including age and villages. ^2^ Overall P-Value >0.05.

**Table 3:** Multivariate analyses comparing relative risks for colorectal cancer in relation to folic acid supplement intake, using varying criteria for definition of exposure and inclusion of adjustment variables.

Sensitivity analysis	Exposed cases/control (n)	OR (95% CI)^1^ history of folic acid intake vs no intake	OR (95% CI)^1^ ≥5 yrs folic acid intake vs no intake

All exposures recorded at least 36 months since diagnosis	27/61	0.81 (0.46 to 1.42)	1.00 (0.3.7 to 2.65)
Restricted analysis to those with full information on adjusting variables	22/62	0.95 (0.51 to 1.79)	0.81 (0.32 to 2.05)


^1^ Adjusted for smoking, body mass index, hypertension, diabetes, GI disorders, Overall P-value >0.05. **Abbreviations:** CI, Confidence interval.

## Discussion

Maternal folic acid supplementation did not adversely affect colorectal cancer risk in a population where supplemental folic acid is regularly prescribed for women of child-bearing age and wheat flour is the staple food being fortified with this vitamin [21]. The stratification to the duration of supplementation and tumor site gave similar results.

Findings from present naturalistic investigation of real world data are consistent with that of a recent meta-analysis showing null effect of folic acid supplementation on colorectal cancer risk within five years follow-up. The results of 10 European countries, as well as that of Japanese and US populations, demonstrated no significant effect of folate levels in blood on colorectal cancer risk [22,23]. On the contrary, studies in Swedish, Korean, and Chinese populations found high blood levels of folate were associated with increased risk of colorectal cancer [20,24]. These controversial findings, especially from East Asian populations, may support the role of ethnicity, population-specific lifestyle, and dietary habits in modifying the effect of folate or folic acid intake on colorectal cancer risk. However, Gao et al. found protective effects of folic acid supplementation for three years on colorectal adenoma risk in the Chinese population [25]. It is worthwhile to note that subjects were pathologically confirmed to be free of any adenomas before the intervention, had lower baseline folate status, and had lower folic acid dosage prescribed (0.5 mg/week vs. 2.8 mg/week for 39 months throughout pregnancy and weaning period, based on WHO guidelines). Although serum/plasma folate is a representative of folate/folic acid intake, it is not feasible to distinguish folate and folic acid using these biomarkers. Taking into account the difference between folate and folic acid metabolism in the body, and their effect on colorectal cancer, it is crucial to assess the findings on folate status with caution.

It is worthwhile to note that folic acid supplementation in the present study was implemented in specific intervals, which in turn impeded excess folic acid intake. From a pathological view, excess folate might lead to carcinogenesis through pre-cancerous cellular replication, tumor genesis activation, and natural cell killers inhibition due to the crucial role of folate in methylation [26]. The adverse effect of excessive folic acid intake gets intensified in colorectal cancer because of high replication rates in colorectal epithelial cells and higher folate absorption, likewise due to excess folate production by colorectal microbes [27]. As a support of the latter, Cole et al., in a trial in the US [28], reported an adverse effect of folic acid supplementation for seven years on colorectal adenoma recurrence. Consequently, supplementation—with dosage and intervals recommended by WHO—for women of childbearing age might prevent the latter adverse effects.

Given the long period of data collection time, the potential confounding variables were sufficiently followed up. Nevertheless, we studied the time interval between folic acid intake and colorectal cancer up to 36 months, and likewise the majority of studies had a short follow-up time. Since the plausible time interval between folic acid intake and incidence of colorectal cancer is proposed to be >10 years, further investigations with longer follow-up time are required to reveal any potential risk of folic acid supplementation on colorectal cancer in this population. Even though studies in the US population demonstrated beneficiary effects of high folate intake with time lag of >12 years [29], as well as that of high folate before and after fortification [30], scarce findings from other nations exist on the effect of folic acid supplementation with plausible time lag. Another issue is that in our study, limited cases had history of folic acid supplement intake before the fortification period (5 cases). Consequently, it was not possible to investigate the association stratified for before and after fortification.

This study is among the investigations assessing the association between folic acid supplement intake and colorectal cancer risk in developing nations. Due to local culture, we were not able to extract valid data for alcohol consumption, data on dietary fiber, or processed meat consumption. In addition, precise estimations of physical activity were not available for the study population. Nonetheless, these factors are expected to be distributed evenly among the rural population, and thus between cases and their matched controls. Also, as our study population consisted of women from rural parts of a province with a very small percentage with history of smoking, it was not possible to assess the risk of folic acid supplementation in the smoking subgroup. Furthermore, we had a limited number of cases and were restricted to women, although all inhabitants of rural parts diagnosed with colorectal cancer were included. Men were excluded since the complete data regarding supplement intake was not recorded for them. Moreover, it was not possible to assess folate intake and folate levels in blood, and yet the database was unique, actively recording the data on supplementation and other health related factors in women of child-bearing age.

The findings of present investigation demonstrated that maternal folic acid supplementation did not adversely affect colorectal cancer risk in a population where supplemental folic acid is prescribed with regular intervals for women of child-bearing age.
